# Supplementary material for: Torque Teno Sus Virus 1: A Potential Surrogate Pathogen to Study Pig-Transmitted Transboundary Animal Diseases
Source: Viruses. 2024 Aug 31;16(9):1397. doi: 10.3390/v16091397 (PMC11436127; doi:10.3390/v16091397)
Supplement: Supplementary file 1 [file viruses-16-01397-s001.zip › Table S3 and Figure S1.pdf]

Table S3. Comparison of three molecular clock models used in BEAST.

	Molecular clock model	Particle # in nested sampling	Marginal likelihood	Standard deviation
1	Strick clock	30	-2973.39	1.97
2	Random local clock	30	-2973.44	1.97
3	Optimized relaxed clock	30	-2971.08	1.93

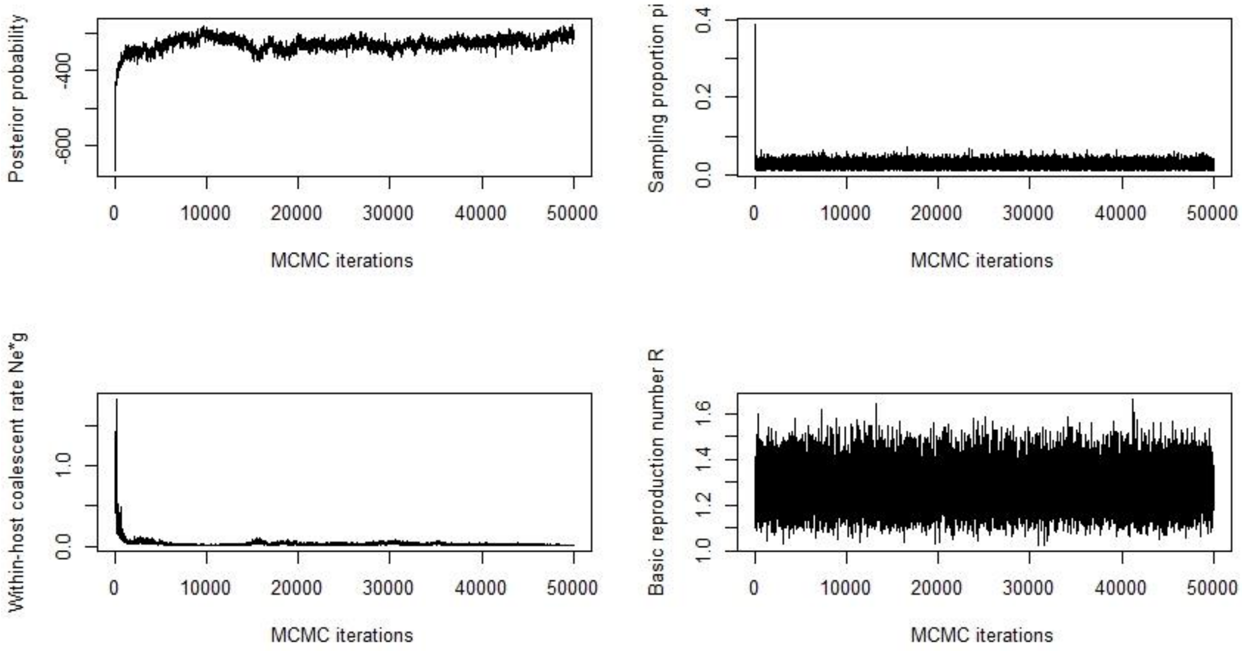

Figure S1. Trace plot of model parameters through MCMC iterations used in TransPhylo.
